# Divergent roles of PD-L1 in immune regulation during ischemia–reperfusion injury

**DOI:** 10.3389/fimmu.2022.1021452

**Published:** 2022-11-21

**Authors:** Jianheng Luo, Ke Liu, Yong Wang, Hongge Li

**Affiliations:** Department of Neurology, Union Hospital, Tongji Medical College, Huazhong University of Science and Technology, Wuhan, Hubei, China

**Keywords:** PD-L1, ischemia–reperfusion injury, costimulatory molecules, immune response, immune checkpoints

## Abstract

Ischemia–reperfusion (I/R) injury is a type of pathological injury that commonly arises in various diseases. Various forms of immune response are involved in the process of I/R injury. As a member of the B7 costimulatory molecule family, programmed death 1-ligand 1 (PD-L1) is an important target for immune regulation. Therefore, PD-L1 may be implicated in the regulation of I/R injury. This review briefly describes the immune response during I/R injury and how PD-L1 is involved in its regulation by focusing on findings from various I/R models. Despite the limited number of studies in this field of research, PD-L1 has shown sufficient potential as a clinical therapeutic target.

## Introduction

Ischemia–reperfusion (I/R) is a heterogeneous pathological condition that is involved in the injury process of various organs. I/R injury plays an important role in cardiovascular and cerebrovascular diseases, which are the leading causes of death worldwide (1, 2). Ischemia is defined as the restriction of blood supply to one or multiple organs. In general, restoring blood flow is the most effective strategy for treating ischemia. However, the process of restoring blood flow can also induce injury. I/R injury is the term used to describe this phenomenon, whereby secondary injury is caused by blood flow restoration to ischemic tissues (3). The most common cause of I/R injury is thrombosis. Circulatory system disorders [e.g., circulatory arrest and sickle cell disease (4)], obstructive sleep apnea (5), and so on can also induce I/R injury.

Although ischemic injury is a relatively simple process to understand, reperfusion injury is more complex and its underlying mechanisms remain unclear. Typically, the I/R injury process is subdivided into six participating components, including vascular leakage, cell death programs, transcriptional reprogramming, autoimmunity, innate and adaptive immune activation, and the no-reflow phenomenon (6). The immune response plays an important role in the I/R injury process.

Programmed death 1 (PD-1)-ligand 1 (PD-L1), also named B7-H1 or CD274, is the third member of the B7 family, a costimulatory receptor family, which belongs to the immunoglobulin superfamily. PD-L1 is a type I transmembrane protein composed of 290 amino acids, with immunoglobulin V-like and C-like domains, a hydrophobic transmembrane domain, and a cytoplasmic tail that is 30 amino acids in length. In humans, PD-L1 is encoded by the *CD274* gene located on chromosome 9 (7, 8).

As the ligand of PD-1, PD-L1 is best known for its role in immune checkpoint inhibition, which is important in immune regulation by bidirectional intracellular signaling between immune cells and target cells. Since its discovery in 1999, PD-L1 has been extensively researched within the cancer field. Nowadays, inhibitors of PD-L1, such as durvalumab and avelumab, are used in targeted cancer immunotherapy in the clinic.

In addition to tumor immunity, an increasing number of studies have confirmed that PD-L1 is also involved in autoimmunity, infection, I/R, and other pathological injuries. Cancer and cardiovascular and cerebrovascular diseases are the two leading causes of adult deaths worldwide. The proportion of patients with cardiovascular and cerebrovascular diseases who are receiving PD-L1-targeted therapy may increase in the near future with the widespread application of this form of therapy in the clinic. It is therefore imperative to explore the role of PD-L1 in I/R injury. Contrary to its popularity in oncology, only a few studies of PD-L1 focus on I/R injury even though the immune mechanisms involved in I/R injury have been confirmed (6). Thus, in this review, we 1) briefly discuss the current understanding of the immune response during I/R injury, 2) summarize the existing research on PD-L1 in this process, and 3) propose potential directions for future research in this field.

## Immune response in ischemia–reperfusion injury

**Figure 1:**
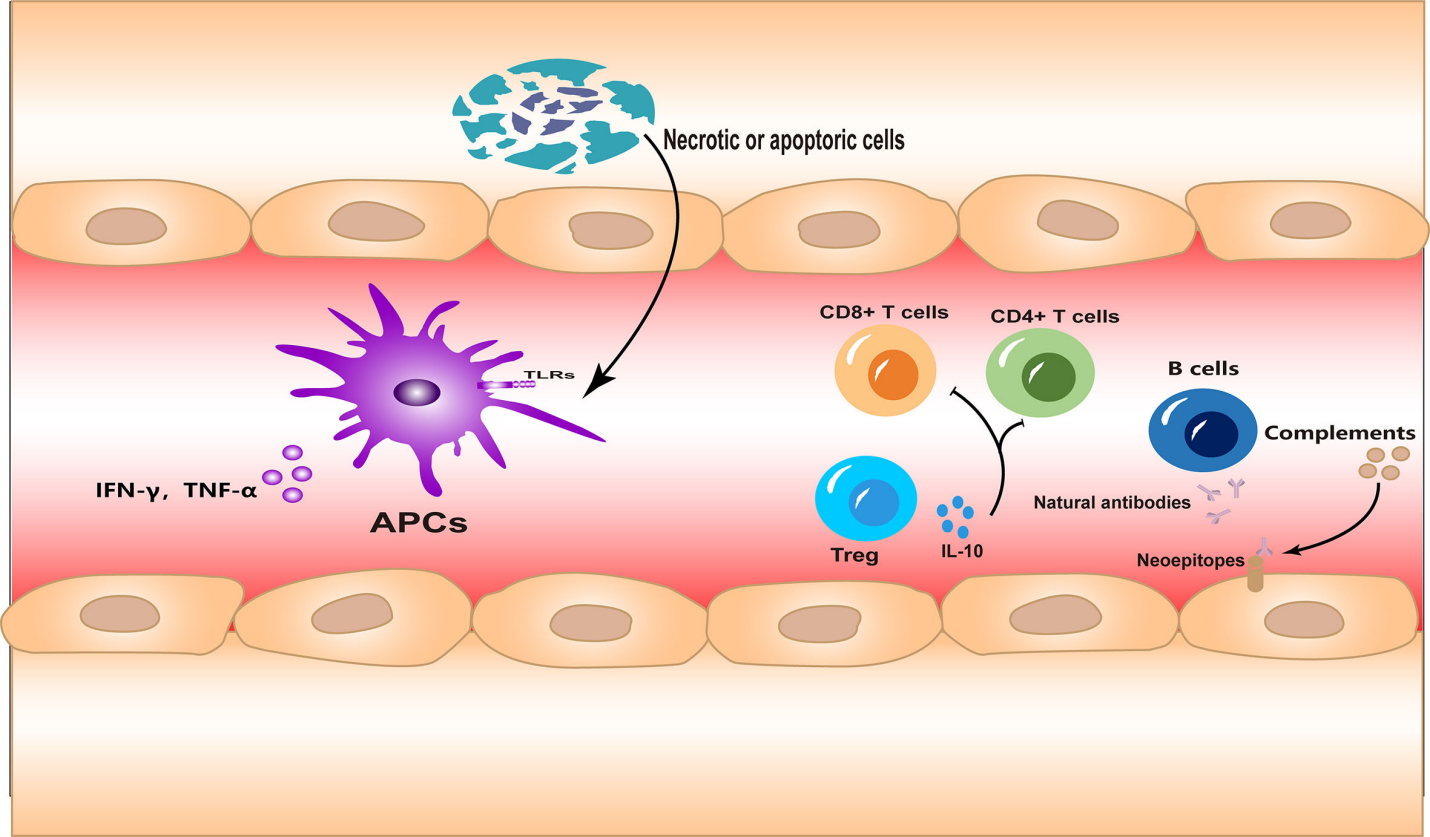
The innate and adaptive immune responses and autoimmunity are all involved in ischemia–reperfusion (I/R) injury. Necrotic or apoptotic cells can directly activate the innate immune response during I/R injury. Different subsets of lymphocytes are involved in the adaptive immune response in different forms. Meanwhile, neoepitopes are exposed to injured tissues, inducing autoantibody production and complement system activation.

The immune system in I/R injury is a complex process, and there are various distinct types of immune response involved. In this section, we will briefly introduce how three aspects of immunity are involved in I/R injury (**Figure 1**).

### Innate immune response

It is important to clarify that, except for a few cases, such as intestinal injury or septic shock, I/R injuries occur in a sterile environment. This means that during I/R injury, the inflammatory response is always in the form of sterile inflammation. In the early stages of I/R injury, large numbers of cells die of necrosis due to hypoxia. Necrotic cells are highly immunostimulatory and lead to inflammatory cell infiltration and cytokine production (9). Subsequently, ligands associated with processes such as cell damage or death, which are termed “damage-associated molecular patterns” (DAMPs), are released or upregulated. These DAMPs bind to innate immune receptors such as Toll-like receptors (TLRs), which activate the innate immune response (10, 11). This process has many similarities to that initiated under non-sterile conditions. Meanwhile, oxidative stress during reperfusion leads to the upregulation of TLR4, which may be another factor that enhances the innate immune response during I/R injury (12).

On the other hand, the accumulation of innate immune cells, which are the main type of infiltrating cells during early injury, also occurs during I/R. The various types of infiltrating innate immune cells play important roles in the removal of necrotic material and tissue repair (13). However, excessive infiltration of immune cells can further exacerbate inflammation (14, 15).

### Adaptive immune response

In contrast to what is known about the immune response in I/R injury, it is not clear how the adaptive immune response is activated under sterile conditions. It is likely that both antigen-specific and antigen-independent mechanisms of activation are involved (16, 17). Studies of the brain (18, 19), heart (20), liver (17), and kidney (21) have indicated that adaptive immune cells infiltrate and participate in the injury process during I/R. The adaptive immune response is even more complex and sophisticated than the innate immune response. In adaptive immunity, diversity arises from different subgroups of immune cells and the intricate combinations of receptors, ligands, and signaling molecules. Studies have shown that CD4^+^ and CD8^+^ T cells primarily mediate the detrimental responses in I/R (17, 19–21), while regulatory T cells (Tregs) offer a protective role (22).

### Autoimmunity

In addition to the two branches of immunity described, autoimmunity should not be ignored. Self-reactive innate recognition proteins can target the neoepitopes expressed in ischemic tissues and initiate inflammation (23). Indeed, the existence of an autoimmune response to I/R injury, mediated by natural antibodies, has been confirmed in several studies (24, 25). Antibodies can in turn activate the complement system, further amplifying the inflammation (26). In animal models of I/R, therapeutic agents targeting the complement system have shown promise (27).

## Expression and regulation of programmed death 1-ligand 1

Considering the complexity of PD-L1 and the limitations of our existing understanding of its function, we will first review our knowledge on PD-L1 expression and regulation mechanisms. Unlike PD-1 and PD-1-ligand 2 (PD-L2), which are only expressed by hematopoietic cells, PD-L1 is also found in a wide range of non-hematopoietic cells, such as vascular endothelium, keratinocytes (28), and epithelial tubular cells (29, 30).

As previously mentioned, PD-L1 is encoded by the *CD274* gene, located on chromosome 9p24.1 in humans, which is also the site of the *JAK2* and PDCD1LG2 (encoding PD-L2) genes. It is worth noting that *JAK2* can encode a protein called Janus kinase 2 (JAK2) that upregulates the expression of PD-L1 mRNA *via* the JAK2–signal transducer and activator of transcription (STAT) signaling pathway (31, 32). In addition, PD-L1 overexpression can be disrupted by Clustered regularly interspaced short palindromic repeats/CRISPRassociated protein 9 (CRISPR/Cas9) technology by targeting the 3′-untranslated region (UTR) of the *CD274* gene (33). In addition to targeting DNA, the modification of histones is another strategy for regulating PD-L1. Some studies have already proven that altering the acetylation or methylation of histones can enhance PD-L1 mRNA expression (34, 35).

MYC, an oncogenic transcription factor, binds the *CD274* promoter and triggers the expression of PD-L1 (36). This phenomenon occurs in many types of tumors. Moreover, inflammation is also strongly correlated with PD-L1 expression. Interferon (IFN)-γ, a pro-inflammatory cytokine, may be the most prominent soluble inducer of PD-L1 expression. After binding to its receptor, IFN-γ promotes the expression of PD-L1 *via* the JAK-STAT signaling pathway (37). Transforming growth factor (TGF) β, TLRs, and various interleukins (ILs) are also involved in the regulation of PD-L1 expression. In addition, there are many other mechanisms including hypoxia-inducible factor-1 (HIF), phosphatidylinositol 3-kinase-Akt/mechanistic target of rapamycin (PI3K-Akt/mTOR), the nuclear factor κB (NF-κB), and mitogen-activated protein kinase (MAPK) signaling pathways.

Aside from these better-known mechanisms, recent studies have discovered new possible mechanisms regulating PD-L1 expression. Liver kinase B1 (LKB1) can increase PD-L1 expression *via* the Kelch-like ECH-associated protein 1/Nuclear factor E2-related transcription factor (KEAP1/NRF2) axis (38). A study on Non-small cell lung cancer (NSCLC) suggested that AMP-activated protein kinase (AMPK) can downregulate LKB1 expression to reverse its function. Another study of lung cancer found that Yes-associated protein (YAP) and PD-L1 expression were positively correlated (39). Furthermore, a study of endometrial cancer used co-immunoprecipitation to show the direct interaction between PD-L1 and AMPK (40). These new findings may help further clarify the mechanism of PD-L1 expression and regulation.

After translation, PD-L1 undergoes several modifications, including N-linked glycosylation, serine/threonine and tyrosine phosphorylation, polyubiquitination, and palmitoylation. N-linked glycosylation is the most notable of the PD-L1 modifications; it has two primary functions: 1) stabilizing PD-L1 and 2) regulating PD-L1/PD-1 binding (41, 42).

In addition to the membrane-bound forms of PD-L1 (mPD-L1), PD-L1 can also be secreted in the form of soluble and/or exosomal proteins (34). Early studies have shown that the production of soluble forms of PD-L1 (sPD-L1) is related to the mPD-L1 expression. mPD-L1^+^ myeloid-derived immune and tumor cells may be the major source of sPD-L1 (43), while matrix metalloproteinases (MMPs) might be involved in the process by which sPD-L1 is produced. MMPs can cleave the extracellular fraction of mPD-L1, releasing sPD-L1 (43, 44). Although mPD-L1 can be expressed in activated T cells, the available evidence suggests that these cells are not involved in the formation of sPD-L1 (43, 45). Recently, exosomal PD-L1 has come into the spotlight, as it was found to be expressed by various cancer types including metastatic melanoma, breast cancer, and head and neck squamous cell carcinoma (37, 46, 47). In addition, exosomal PD-L1 can induce osteogenic differentiation and promote fracture healing through its immunosuppression ability (48).

## The programmed death 1/programmed death 1-ligand pathway

**Figure 2:**
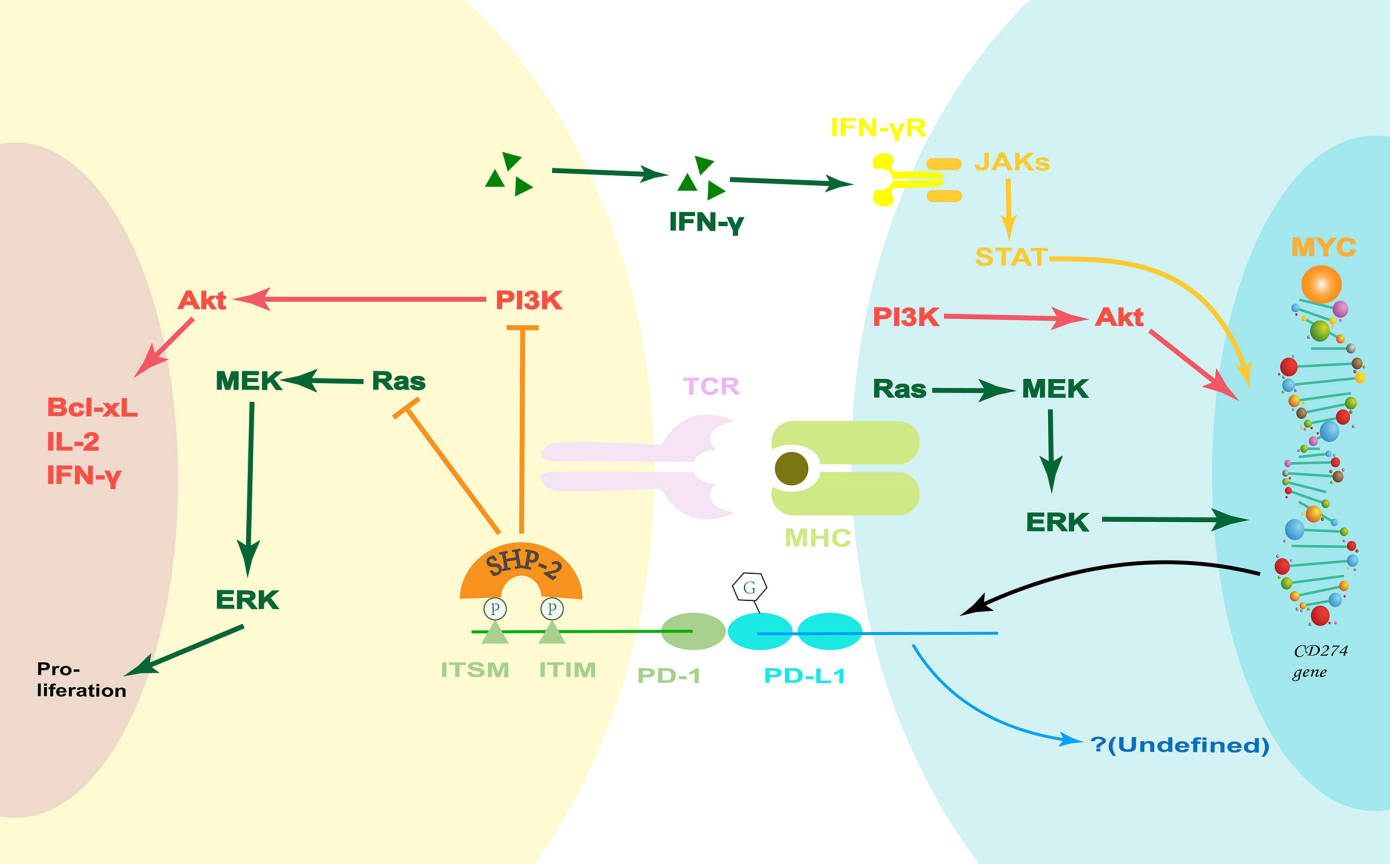
The PD-1/PD-L1 pathway. PD-1 ligation inhibits the PI3K-Akt and Ras-MEK-ERK signaling pathways. Coincidentally, the PI3K-Akt and Ras-MEK-ERK pathways are upstream signals that regulate PD-L1 expression. Moreover, IFN-γ can increase the PD-L1 expression *via* the classical JAK-STAT pathway during inflammation. However, the potential PD-L-associated downstream signaling pathways are undefined, and they may hold the key to how the PD-1/PD-L1 pathway regulates the innate immune response. Bcl-xL, B-cell lymphoma-extra large; ERK, extracellular signal-related kinases; IFN, Interferon; ITIM, immunoreceptor tyrosine-based inhibitory motif; ITSM, immunoreceptor tyrosine-based switch motif; JAK, Janus kinase; MEK, mitogen-activated extracellular signal-regulated kinase; MHC, major histocompatibility complex; PD-1, Programmed death 1; PD-L1, PD-1-ligand 1; PI3K, phosphatidylinositol 3-kinase; SHP-2, Src homology 2 domain-containing protein tyrosine phosphatase-2; STAT, JAK2 signal transducer and activator of transcription; TCR, T-cell receptor.

PD-L1 is one of two ligands that bind PD-1. It is therefore important to mention the PD-1/PD-L pathway. From the perspective of structural biology, PD-L2 has a higher affinity for PD-1 than that of PD-L1 (49). However, the general abundance of PD-L2 is much lower than that of PD-L1. Therefore, PD-1/PD-L1 is the dominant signaling pathway. When PD-1 bind to its ligands, Src homology 2 domain-containing protein tyrosine phosphatase-2 (SHP-2) is recruited *via* the phosphorylated immunoreceptor tyrosine-based inhibitory motif (ITIM) and immunoreceptor tyrosine-based switch motif (ITSM) tyrosine motifs of PD-1 (50, 51). This leads to the attenuation of the PI3K-Akt (52, 53) and Ras-extracellular signal-related kinases mitogenactivated extracellular signal-regulated kinase (ERK-MEK) (52) signaling pathways, decrease in the production of certain cytokines (e.g., IL-2 and IFN-γ), and inhibition of cell cycle progression (**Figure 2**).

In contrast, on the other side of the costimulatory pathway, there is some evidence that PD-Ls can also transmit a costimulatory signal. For example, in a study using sPD-1, it can be observed that the phenotypic changes of dendritic cells (DCs) are related to the activation of the PD-1/PD-L pathway (54). However, the potential PD-L-associated downstream signaling pathways have not been fully studied. It should be noted here that both PD-1 and PD-Ls can be expressed on antigen-presenting cells (APCs) and lymphocytes. At present, the role of PD-1 expression on APCs is not clear.

Formerly, it was believed that the PD-1/PD-L pathway was only involved in the regulation of the adaptive immune response. However, new research suggests that it is also implicated in the innate immune response (55–59) and autoimmunity (54, 60). The PD-1/PD-L signaling pathway can affect the infiltration of monocytes/macrophages and neutrophils, as well as regulate the function of DCs and nature killer (NK) cells. This suggests that the expression of PD-1 and PD-L1 on APCs may be involved in their regulation of innate immune response, which is a potential direction for future research.

## Pleiotropic effects of programmed death 1-ligand 1 in ischemia–reperfusion injury

There are many links between PD-L1 and I/R injury. Both sterile inflammation and immune activation are involved in the I/R injury process. Since PD-L1 serves as an immune checkpoint molecule and is regulated by markers of inflammation, there is plenty of reason to assume that PD-L1 plays an important role in I/R injury (**Table 1**).

**Table 1:** PD-L1 in I/R models.

Expression	The Form of I/R	Effects	Involved Immune Cells	Refferences
Up	OHCA	–	Lymphocytes(Periphral Blood)	[61]
Human	ASC**	–	CD4+CD25+Foxp3+Tregs	[64]
Up	Warm Ischemia(90min)/Reperfusion(6h)	Prevent hepatocellular damage;	T cells, Neutrophils, Macrophages	[80]
		Attenuate lymphocytes infiltration;		
		Inhibit I/R-induced liver necrosis/apoptosis		
Up	Cold Ischemia(24h,4℃)/Reperfusion(1,3,6 or 12h)	Prevent hepatocellular damage;	CD8+T Cells	[81]
		Attenuate lymphocytes infiltration;		
		Regulate the apoptosis of donor CD8+T cells;		
		Down-regulate inflammation-related		
–	Bilateral Renal Ischemia(21,24 or 26min)/Reperfusion(24h)	PD-L1 blocked:	CD11b+ Cells***	[29]
		Exacerbate renal dysfunction and tissue injury;		
		Enhance the innate immune response in the kidney;		
		Lead to increased level of IL-6, CXCL1 and ICAM-1		
Up	Ischemia(35min)/Reperfusion(Left)	–	–	[30]
Down	Ischemia(1h)/Reperfusion(2h)	–	PP CD4+T Cells	[68]
Down	Ischemia(1h)/Reperfusion(2h)	Attenuate intestinal immune dysfunction;	PP CD4+T Cells	[69]
Promote AID expression via IL-10/miR-155 pathway
–	Ischemia(1h)/Reperfusion(24h)	PD-L1 KO:	APCs(Spleen), CD8+CD122+ Tregs	[71]
		Ameliorate infarct volume and reduce neurological deficits;		
		Reduce cerebral cell infiltration and inflammation;		
		Rescue MCAO-induced splenic atrophy;		
		Inhibit activation states of splenic lymohpcytes;		
		Lead to increased expression of PD-1 on T cells and decreased expression of CD80 on APC in spleens;		
		Result in loss of suppressor Tcells from spleens		
–	Ischemia(1h)/Reperfusion(24h)	PD-L1 blocked:	APCs(Spleen), CD8+CD122+ Tregs	[75]
		Ameliorate infarct volume and reduce neurological deficits;		
		Reduce cerebral inflammation;		
		Enhance the accumulation of CD8+ Tregs in the ischemic hemisphere;		
		Rescue MCAO-induced splenic atrophy;		
		Lead to decreased expression of CD80 on APC in spleens;		
		Increase the expression of regulatory molecules on T cells in spleens		
–	Ischemia(1h)/Reperfusion	Decrease the neutrophil-derived MMP-9	CD4+CD25+ Tregs, Neutrophils	[77]
		Preserve BBB integrity		
		Lead to neuroprotection		

### Programmed death 1-ligand 1 expression is associated with ischemia–reperfusion injury

Changes in PD-L1 expression have been shown to be correlated with I/R injury in multiple models. A clinical study on 30 out-of-hospital cardiac arrest (OHCA) patients indicates that their sPD-L1 plasma levels increased during cardiopulmonary resuscitation (CPR) and were significantly higher than those of healthy volunteers (61). Considering that previous studies have indicated that sPD-L1 is elevated not only in cancer but also in general inflammation (43, 70, 71), other inflammatory disturbances were considered in this study. Although initially thought to be associated with the presence of pneumonia, the sPD-L1 levels in the OHCA group remained higher than those in the healthy control group independently of pneumonia, which is thought to result from I/R injury. Meanwhile, the plasma sPD-L1 levels correlated with certain clinical parameters (e.g., blood urea nitrogen (BUN), estimated glomerular filtration rate (eGFR), and C-reactive protein) and disease progression. This suggests that PD-L1 could serve as a clinical biomarker. Moreover, sPD-L1 levels appeared to correlate with the severity of organ failure, which was indicated by the Sequential organ failure asses (SOFA) scores. Another clinical study involving 70 patients indicated that the expression of PD-L1 on Tregs was significantly lower in patients with acute coronary syndrome (ACS) compared with that of the control group and patients with coronary heart disease (CHD; including patients with stable angina pectoris, silent myocardial ischemia, or ischemic heart failure) (62). No significant difference in Treg-specific PD-L1 expression was observed between these two latter groups. Although this study excluded interference from atherosclerosis, a pathological process in which PD-L1 is known to be involved (72–74), it did not specify whether changes in PD-L1 expression occurred during ischemia or possible reperfusion.

In an experimental kidney C57BL/6 mice model of I/R injury, ischemia was performed for 35 min at 25°C and then blood flow was restored. PD-L1 expression was induced in early injury (on days 3 and 7), whereas renal recovery occurred on day 10 (30). However, in an intestinal BALB/c mouse model of I/R, in which ischemia was induced for 1 h, followed by a 2-h reperfusion, the PD-L1 expression on Peyer’s patches (PPs) CD4^+^ T cells was markedly decreased. Meanwhile, the levels of protective soluble immunoglobulin A (sIgA) in the lavage fluid were also decreased. Moreover, a positive correlation was observed between PD-L1 expression and sIgA levels (65).

### Programmed death 1-ligand 1 showed inconsistent effects in the ischemia–reperfusion models of different organs

Whether PD-L1 is involved in the progression of I/R injury and its specific function in this process has not been clarified. Multiple I/R models of different organs have produced inconsistent results.

Using a recombinant PD-L1 Fc protein in an intestinal I/R model further proved that PD-L1 Ig lowered PD-1 expression and intestinal immune dysfunction *via* the IL-10/miR-155 pathway (66). However, unlike the anti-IL-10 monoclonal antibody (mAb), using the miR-155 Agomir did not reverse the protective effect of PD-L1 Ig, which may suggest that there are other potential mechanisms downstream of IL-10 aside from miR-155. This study suggests the possibility of using recombinant PD-L1 Fc proteins in the clinical treatment of intestinal I/R injury. It may be used as a prophylactic medication before intestinal surgery.

In a kidney I/R injury model, PD-L1 also seemed to show a protective function. Blocking either PD-1 ligand 24 h prior to I/R injury (21-min or 24-min or 26-min ischemia followed by 24-h reperfusion of bilateral kidneys) increased organ injury, dysfunction, and inflammation (29). Moreover, blocking both PD-L1 and PD-L2 further exacerbated I/R injury in comparison with blocking either one alone. This conclusion was reinforced in a mild renal I/R injury model using PD-L1 or PD-L2 knock-out (KO) mice, in which ischemia was induced for 21 min followed by a 24-h reperfusion. The main infiltrating cells in this research model were innate immune cells, and blocking of PD-1 ligands showed no effect on CD4^+^ and CD8^+^ T-cell infiltration. Since PD-L1 expression is not restricted to immune cells, the researchers also investigated the contribution of PD-L1 in the immune and non-immune compartments by generating bone marrow (BM) chimeric mice. The result showed that there was no significant difference in renal function between the wild-type (WT) recipient (whether they belong to the sham group or I/R injury group), their BM donor from WT mice, or PD-L1 KO mice. This means that PD-L1 on non-BM-derived cells (BMDCs), but not on BMDCs, protects the kidney from the I/R injury.

The study of the central nervous system (CNS) is further complicated by the existence of the blood–brain barrier (BBB) (75). Halina Offner and her team conducted a series of studies on the role of the PD-1/PD-L1 costimulatory pathway in experimental stroke (67, 68, 76). In their experimental stroke model, WT C57BL/6J mice or PD-L1 KO mice were subjected to 60 min of middle cerebral artery occlusion (MCAO), followed by 96 h of reperfusion. Surprisingly, the lack of PD-L1 reduced CNS inflammation and infarct volume (67). They also observed decreased total cerebral inflammatory cell infiltration. Especially, the number of CD8^+^ T cells significantly increased not only in the ischemic right hemispheres but also in the non-ischemic left hemisphere of the brain. Furthermore, the researchers also observed that PD-L1 knockdown rescued MCAO-induced splenic atrophy and decreased the expression of CD80 [also named B7-1, another costimulatory molecule that interacts with PD-L1 (77–79)] on splenic APCs. Based on these results, they used an anti-PD-L1 mAb to treat ischemic stroke, further proving that PD-L1 is a negative factor in ischemic stroke (68). Not only do these results contradict conventional wisdom, but they also contradict some other studies. In a previous study, the researchers discovered that the increased expression of PD-1 limits CNS inflammation (76). In another study, PD-L1 played an essential role in Treg-afforded protection against BBB damages after stoke (69). Professor Offner and her team attribute these disparate results to CD8^+^CD122^+^ Tregs (67, 80). Although studies of mouse cell lines deficient in specific populations of lymphocytes showed that CD8^+^ T cells have a detrimental function in the I/R injury of the brain, Tregs are protective factors (19–21). Their research showed that the CD8^+^CD122^+^ Tregs accumulated in the damaged side of the brain following stroke. In addition, heterogeneous binding between costimulatory molecules may be another factor. It appears that after MCAO, the absence of PD-L1 causes an increase in the expression of PD-L2 and a decrease in the expression of CD80 on splenic APCs. Although these factors can partly explain the contradictory results, the specific mechanism has not been systematically elucidated.

### Programmed death 1-ligand 1 in ischemia–reperfusion injury during organ transplantation

Organ transplantation is a unique case in which the genetic composition of immune cells and target cells are mismatched. This means not only that I/R injury during transplantation is accompanied by a more complex and stronger immune response but also that the expression of PD-L1 on immune cells (mainly recipient-derived) and target cells (mainly donor-derived) during transplantation is different from that under normal conditions.

It was previously believed that there was no correlation between the upregulation of PD-L1 expression and I/R injury during transplantation (81). However, new research contradicts this conclusion. In the studies of liver transplantation, the expression of PD-L1 protected the transplanted liver and limited I/R injury in both the cold I/R model and warm I/R model (63, 64). In the cold I/R model, a chimeric liver was used to show that the expression of PD-L1 on hepatocytes and BMDCs of the donor liver was equally important (64). This contradicts the results of the renal I/R study we mentioned earlier (29). In addition, a significant increase in both the host-derived and graft-derived CD8^+^ T cells was observed in the cold I/R model. Meanwhile, neutralization of IL-10 reversed the protective effect of PD-L1 in the warm I/R model (63). This evidence suggests that the PD-L1 function is T cell-dependent, which is consistent with the traditional viewpoint and the conclusion of the aforementioned intestinal I/R model study (66).

## Programmed death 1 and programmed death 1-ligand 2 in ischemia–reperfusion injury

Although PD-L1 is the focus of this review, it is important to mention PD-1 and PD-L2 in the context of I/R injury, as these molecules are closely related to PD-L1 function.

A study on renal I/R injury has suggested that the protective effect of the adenosine 2A receptor in acute kidney injury (AKI) may be due to the increased expression of PD-1 on Tregs (82). Two studies on ST-elevation myocardial infarction also identified a correlation between PD-1 and I/R injury (83, 84). By analyzing peripheral blood mononuclear cells (PBMCs), they observed a sharp increase in PD-1 expression before reperfusion and a significant decrease after reperfusion. Moreover, the decrease in PD-1 expression on both PBMCs and in the infarct areas was associated with extensive infarction. In the MCAO model, the expression of PD-1 on immune cells also increased significantly, and PD-1 deficiency exacerbated infarct volume and inflammation of the CNS (76). These findings highlight the protective effect of PD-1 in the context of ischemic stroke. Subsequent research implied that the protective effect of IL-10 may be partly mediated *via* the increase in PD-1 expression on lymphocytes (85).

Less is known about the role of PD-L2 in the pathology of I/R injury. In the aforementioned study of kidney I/R, the researchers observed that PD-L2 also showed a protective effect, alongside PD-L1 (29). In the experimental stroke model, PD-L2 KO had a similar effect to that of PD-L1 KO (67). However, the intracranial infiltrating immune cell subsets differed between the PD-L1 KO and PD-L2 KO mice. Another study involving vessel transplantation discovered that PD-L2 expression was increased independent of IL-1β and IL-18 levels (86). Recently, some researchers have shown that the simultaneous activation of the PD-L2- and Cytotoxic T Lymphocyte antigen 4 (CTLA-4)-related pathways with a novel human fusion recombinant protein improved renal function in both the conventional and allograft models of renal I/R (87). This may indicate the potential of PD-L2 as a clinical therapeutic target. However, it should be noted that this study did not compare the effects of PD-L2 or CTLA-4 activation alone.

## Discussion

PD-L1 is a coinhibitory molecule belonging to the B7 family (7, 28, 88). Studies of I/R models have long suggested that the immune response plays an important role in the pathology of I/R injury. However, due to the complexity of the immune system and the limitations of our existing understanding, it is still unclear what position PD-L1 holds in I/R injury. PD-L1 is now thought to be involved not only in the adaptive immune response but also in the innate immune response (55–59) and autoimmunity (54, 60). In addition, PD-L1 also has been observed to promote the immune response in some studies (54, 60). In this review, we focused on summarizing some studies of PD-L1 in I/R injury. Although few studies on PD-L1 in the context of I/R exist, their results are contradictory. For instance, they report that during I/R, PD-L1: 1) is either upregulated or downregulated, 2) promotes or limits inflammation, 3) increases or decreases leukocyte recruitment, and 4) implicates either BMDCs or non-BMDCs (61–69). Of course, we could attribute these differences to differences between experimental conditions, materials, methods, or animal models. For instance, with regard to the renal I/R models, although both studies used C57BL/6 mice, the durations of ischemia induction and reperfusion were different (29, 30). In addition, one of these models was bilateral, while the other was performed on the left kidney only. The intestinal I/R model used BALB/c mice (65, 66), while, in the liver transplantation model, the induction of warm or cold I/R was the key variable (63, 64). In the model of ischemic stroke, although the ischemic time was consistent, the time of reperfusion was not. Moreover, in the renal models, the infiltrating immune cells were primarily innate immune cells (29), which could be attributed to the short reperfusion time. However, it is not rigorous to explain these discrepancies without considering the diverse experimental conditions. How the experimental conditions affect PD-L1 has not been fully explained, which is exactly what we need to further explore.

One point that we need to be aware of is that the CNS is an exception to the norm. Although there are many inconsistencies in the aforementioned studies, it is generally believed that PD-L1 has a protective effect on I/R injury in organs such as the liver, the kidney, and the intestine. In contrast, the absence of PD-L1 ameliorated the I/R injury in the CNS (67, 68). The immunologically privileged status of the CNS may explain these differences in the role of PD-L1 in I/R (89, 90). Stroke is often accompanied by BBB damage, which has been demonstrated in several studies (91–94). This damage leads to the exposure of antigenic determinants, inducing the infiltration of immune cells. This may partly explain the increased infiltration of CD8^+^ T cells into the healthy hemisphere of the brain. Necrosis at the injured site causes local inflammation, which leads to the recruitment of more immune cells and increases the expression of PD-L1, thus obstructing the infiltration of CD8^+^CD122^+^ Tregs. In an earlier study of experimental autoimmune encephalomyelitis (EAE), it was discovered that the increased expression of PD-L1 inhibits the infiltration of CD8^+^ Tregs and aggravates the inflammatory response of the CNS, which is consistent with the phenomenon observed in MCAO (54). Although various costimulatory molecules are widely expressed by BMDCs, their expression and function in specific cell subsets are still inconsistent (28, 29). This may be the reason why PD-L1 has a more significant effect on CD8^+^CD122^+^ Tregs.

In renal models using chimeras, PD-L1 expression on non-BMDCs but not on BMDCs was observed to play a role in I/R injury (29). In the liver transplantation model, PD-L1 expression on graft-derived BMDCs and non-BMDCs in grafts showed similar effects (64). This was consistent with the conclusion of the kidney I/R model, considering that the recipient-derived immune cells are the major factor in this immune response. Since PD-L1 is extensively expressed on many cell types, it is worth considering whether the expression of PD-L1 on non-BMDCs plays a different role from that found on BMDCs.

There are more that should be noted in renal models. The increased expression of PD-L1 has been observed in many AKI models aside from the kidney I/R model (30, 95, 96). In all of these models, the distribution of PD-L1 had localized effects. In these AKI models, the increased expression of PD-L1 on tubular cells was observed in all samples. In contrast, only part of the samples showed PD-L1 expression within the glomerular and endothelial compartments. Moreover, PD-L1 expression on tubular cells positively correlated with serum levels of C-reactive protein and the severity of AKI (95). This finding is consistent with the previous view that the expression of PD-L1 on non-BMDCs (but not on BMDCs) in the kidney plays a protective role. In addition, researchers observed that the number of PD-L1^+^ cells in urine samples also positively correlated with tubular PD-L1 positivity (30), which provided a theoretical basis for PD-L1 as a potential biomarker for evaluating AKI. At the same time, the inconsistent expression of PD-L1 in different compartments of the kidney is also worth pondering. One possible hypothesis is that the tubules may be the main site of inflammatory cell infiltration in the kidney. Moreover, the complement system also regulates the expression of PD-L1 and the infiltration of immune cells. It is possible that PD-L1 can be involved in complement-mediated immune cell infiltration processes.

**Figure 3:**
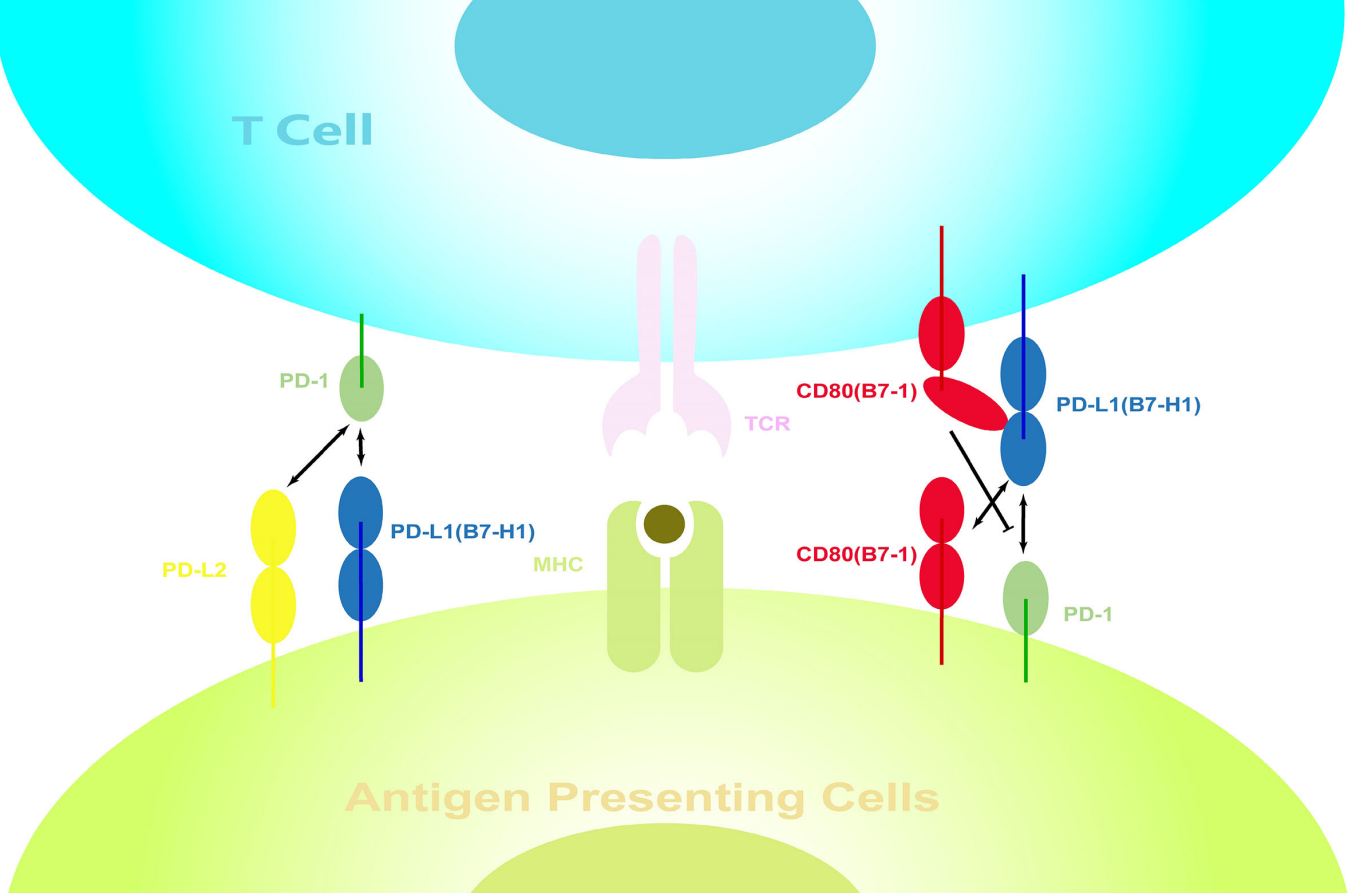
Interactions between costimulatory molecules on the surface of immune cells. Interactions between costimulatory molecules, which are expressed on both APCs and activated lymphocytes, form a complex regulatory network. PD-1 can bind to PD-L1 and PD-L2, while PD-L1 can also bind to CD80. However, the cis-PD-L1/CD80 interactions restrict the PD-1 function.

Another point worth discussing is the nonspecific binding between costimulatory molecules, which we briefly mentioned earlier. We have previously described that PD-L1 plays a superior role to that of PD-L2 in PD-1-mediated signaling. However, on knocking out PD-L1, the situation changed. After MCAO, the loss of PD-L1, together with an increase in PD-L2, leads to the PD-1/PD-L2 becoming the main negative regulatory signaling pathway. Alternatively, PD-L1 can also bind to CD80 (77–79), which is generally regarded as another negative regulatory interaction. However, a series of recent studies have indicated that the cis-PD-L1/CD80 interaction could disrupt PD-1/PD-L1 binding and abrogate the PD-1-mediated inhibitory effects (97–99) (**Figure 3**). Meanwhile, decreased CD80 expression on splenic APCs was observed in PD-L1(-/-) mice, suggesting that the regulation of the expression of these costimulatory molecules may not be independent. There may therefore exist a yet unknown mechanism to coordinate their expression.

In intestinal and experimental stroke models, PD-L1 has shown its potential in the treatment of I/R injury, although their treatment strategies are different. Clinically, the use of PD-L1 recombinant Fc protein has not been approved. In contrast, there are two approved inhibitors of PD-L1. Immune-related adverse events (iRAEs) are noteworthy when considering clinical treatment targeting PD-L1. The iRAE of patients using immune checkpoint inhibitors (ICIs) can involve any organ system (100). Specifically, the most common iRAE in patients using durvalumab is pneumonia (101), and thyroid disease is common in patients receiving avelumab (102). However, it should be noted that these results are based on clinical cohort studies of cancer patients. Considering the tumor microenvironment, cancer patients may have different responses compared with other patients.

In conclusion, our understanding of the effects of PD-L1 in I/R and even PD-L1 itself is still limited. However, both the clinical needs and research findings highlight the potential of PD-L1 as a target in the treatment of I/R injury. Meanwhile, the in-depth study of diverse roles of PD-L1 in I/R is bound to be an excellent springboard for us to further understand PD-L1 and the immune response.

## Author contributions

JL and KL conceptualized the review and drafted the manuscript. HL and YW restructured and revised the manuscript. All authors read and approved the final version of the manuscript.

## Funding

This work was supported by a grant from the National Natural Science Foundation of China (Grant No. 81801181).

## Acknowledgments

We would like to thank Charlesworth Author Services for English language editing.

## Conflict of interest

The authors declare that the research was conducted in the absence of any commercial or financial relationships that could be construed as a potential conflict of interest.

## Publisher’s note

All claims expressed in this article are solely those of the authors and do not necessarily represent those of their affiliated organizations, or those of the publisher, the editors and the reviewers. Any product that may be evaluated in this article, or claim that may be made by its manufacturer, is not guaranteed or endorsed by the publisher.
